# Supporting Young Dads’ Journeys through Fatherhood

**DOI:** 10.1017/S1474746415000524

**Published:** 2016-01

**Authors:** Jessica Cundy

**Affiliations:** National Society for the Prevention of Cruelty to Children E-mail: Jessica.Cundy@NSPCC.org.uk

**Keywords:** Young fathers, voluntary sector, policy, family support, children

## Abstract

While the recent Coalition government committed to some initiatives supporting the role of parents, relationships and the early years, there remains a lack of focus on fathers as a distinct policy area. This is reflected at local government level, as lead professionals for young fathers are rare and data on the number of young fathers in each local area are not routinely collected. Barnardo's was funded by the Department for Education in 2012, as part of the Family Strategic Partnership, to highlight the needs and experiences of young fathers in England, and the joint role of statutory and voluntary services in supporting them (fully reported by Barnardo's in Cundy, 2012). Based on selected case studies drawn from research and a range of practice organisations, this article presents the journeys of five young fathers and their experience of maternity services, children's centres, schools, housing services and the secure estate.

## Introduction

In June 2012, Barnardo's convened a group of leading fathers’ organisations and academics to highlight the needs and experiences of young fathers aged sixteen to twenty-four in England, and the joint role of statutory and voluntary services in supporting them. Taking a life story approach, the project charted the journeys of five young fathers through different areas of service provision. The journeys revealed the blockages that can occur when insufficient support is available, and the opportunities that arise when services respond to young fathers effectively.

The best practice examples emphasise that the most significant change statutory services can make is an attitudinal shift, from focusing solely on the mother and baby to enquiring about the father and what his needs might be. Research has shown time and again that fathers’ engagement with their children, particularly in the early years, can significantly improve outcomes for both them and their children (Flouri and Buchanan, 2004; Carlson, 2006; Sarkadi *et al.*, 2008). There are key points at which intervention with young fathers in particular is proven to be most effective, for example in the period immediately following conception, or while they are still engaged in education (Weinman *et al.*, 2007). The family voluntary sector has an important role to play in working with statutory services to raise awareness of young fathers’ needs, and offer referral routes to the additional support that they may require. This article looks at how mainstream services can tailor the support they provide, so that it better meets young fathers’ needs. It makes the case for an integrated approach to supporting young dads, with support from different agencies brokered through a lead professional within each local authority area.

## Policy context

Both New Labour and the recent Coalition government have made efforts to promote the role of fathers in children's lives. Initiatives such as the Think Fathers campaign^1^ have been supported by fathers’ organisations that recognise the need for all services working closely with mothers to, at the very least, keep fathers in mind. While the Coalition government committed to many initiatives supporting the role of parents, relationships and the early years, there remains a lack of focus on fathers as a distinct policy area. This is reflected at local government level, as lead professionals for young fathers are rare and data on the number of young fathers in each local area are currently not collected.

There have been a number of policies relating to parenting and childcare (particularly in the early years) over the past decade. The introduction of children's centres in the early 2000s created a vehicle for continued and focused interaction with young families for the first time. Parenting and family support remains one of the key services underpinning the core purpose of children's centres as revised in 2012 (Department for Education and Department of Health, 2012). Children's centres have often taken the lead in pioneering ways to engage fathers, such as through Saturday ‘dads’ clubs’ or activity-orientated play days for fathers and children.

Based on evidence from a number of commissioned reviews, recent governments have placed particular emphasis on early intervention as a means to promote better outcomes for children later in life (Field, 2010; Allen, 2011; Tickell, 2011). This underpins much of the Department for Education's policy-making in recent years, including a free early education entitlement for the most disadvantaged two-year-olds,^2^ vouchers to access £100 worth of universal parenting classes^3^ and subsidised counselling and advice services for new parents to help them adjust to family life.^4^ Arguably, the success of these initiatives depends upon the engagement of fathers as well as mothers, although this does not appear to be reflected in current family policy. The case studies in this report highlight the lack of policy development and service provision that respond to the unique needs and circumstances of young fathers.

## Young fathers’ journeys

Here we outline five young fathers’ journeys as they make the transition to fatherhood. In each journey, the fathers come into contact with different statutory and voluntary services and encounter both barriers and support along the way. Common themes throughout the five journeys are that the young fathers:

• are coping with complex identity changes;
• often experience significant financial hardship;
• require legal advice to maintain contact with their child;
• benefit from relationship support to maintain contact with the mother;
• need parenting advice as much as mothers, but tailored to a male audience.

These journeys illustrate the points in young fathers’ lives at which intervention is most effective in terms of positive outcomes for them and their children, as well as the implications of not intervening, or leaving it too late. They reveal the complex and often challenging relationships between maternal and paternal grandparents, their children and their grandchildren, which can act as barriers to support for young fathers (Neale and Lau Clayton, 2014). Significantly, the journeys highlight what previous research has shown: that becoming a father offers opportunities, not just obstacles (Hirst *et al.*, 2006; Duncan *et al.*, 2010). Statutory services can facilitate access to the range of available support for young fathers, as well as instigating much needed culture change within their services. The voluntary sector is particularly good at helping fathers stay on track with education, employment and training (EET) by addressing the multiple factors that may underlie disengagement (Evans, 2012).

## Young dads and maternity services

The Fatherhood Institute (2010) highlights many of the issues faced by young expectant fathers, where both gender and age can act as barriers to gaining the support they need from health services. In the context of NHS (National Health Service) cuts to maternity services, staff training focused on support for fathers-to-be is rare; in 2012/13, five out of ten English regions saw reductions of up to 15 per cent in maternity units, compared with the previous year (Royal College of Midwives, 2013). While local teenage pregnancy teams are often attuned to young fathers’ needs, universal maternity services may exclude young men due to lack of staff awareness and resources (Pollock *et al.*, 2005). A review of US and UK research studies found young fathers often have limited or no contact with midwives, health visitors and social workers (Bunting and McAuley, 2004). It is perhaps understandable that GPs (general practitioners), midwives and health visitors prioritise mother and baby health. Yet by ignoring the role of the father during pregnancy, they miss a crucial opportunity to support positive outcomes for the whole family (Lloyd, 2010). Previous research (Dunn *et al.*, 2004; Flouri and Buchanan, 2004; Carlson, 2006; Martin *et al.*, 2007) has shown that supporting a positive father–mother relationship where possible has a significant impact on outcomes for children. By assessing young fathers’ needs at this early stage, services equip them to support their children in the long term. Midwives and health visitors are also important referrers to voluntary sector support, but they need to be aware of what services are available in the local area.

## Good practice example

Working with Men is a UK charity that provides support, information and advice to professionals, local authorities and government as well as men and their families. The charity's Expectant Fathers Programme^5^ is an evidence-based course that is delivered in hospitals and children's centres across England. The programme aims to build new fathers’ confidence, particularly in terms of their role, their skills and their ability to support their baby. The programme includes an opportunity to ask questions of an experienced midwife, enabling fathers to engage more actively in the pregnancy and understand that they have an important role to play.

## Case study 1: Nick's journey

Nick was seventeen when he and his girlfriend found out they were going to have a baby. Their relationship broke down during the pregnancy. After his child was born, Nick became withdrawn because his ex-girlfriend and her family would not let him see his daughter. He was referred to the charity Working with Men (WWM) by his sixth-form teacher who saw that he needed support. WWM enrolled Nick on an Expectant Fathers Course which allowed him to meet other young fathers, learn about the practicalities of parenting and get advice from a midwife. Nick became more determined to see his daughter and developed confidence to speak up at meetings about his situation. His support worker carried out an assessment of his needs and realised mediation was needed to enable the two families to talk. The maternal grandparents were resistant, but eventually both families agreed to attend a meeting supported by WWM. The young parents’ families were heavily involved in the situation and became angry and upset with each other during the meeting. Eventually, the maternal grandparents agreed Nick could see the baby at specified times at their house.

Nick and his ex-girlfriend got back together. The maternal grandmother was angry about this and forced her to leave the family home. She and the baby went to stay with Nick and his mum. This caused further tension between the maternal and paternal grandparents. Nick and his girlfriend approached housing services for support and the mother and baby were placed in a mother and baby unit. As Nick was aged under eighteen, he was allowed to visit but not live with them.

Nick was living at home with his parents while his partner was moved to semi-independent housing. She was struggling to cope and began drinking heavily and having relationships with other men which was putting her at risk. She was under social care supervision and there were concerns for her baby. At this time, Nick was seeing his daughter weekly and looked after her at his parents’ house. Social services decided that the baby needed to be taken into care after the mother went missing and was found in a risky situation. The baby became a ‘looked after child’ but stayed with the maternal grandmother, who became her primary carer.

Nick wanted his baby to live with him. Social services felt that he was too immature, was not working regularly and lacked life skills. However, he was looking after her for half of the week without financial support. He was told that his daughter would stay with the maternal grandmother until she is eighteen. WWM helped him find a solicitor for advice.

Nick's daughter is now two-and-a-half. His life has changed dramatically. He attends a young fathers group and has developed a commitment to fathers’ rights issues. He did not finish sixth form but started a training course to become a carpenter and has been employed for over a year. He has legal parental responsibility but only sees his daughter for one overnight stay each week. The involvement of the maternal grandparents has had a significant impact on his engagement with his daughter, and he is still seeking legal advice to get a residence order.

## Recommendations for maternity services

• Maternity services should record the father's details during pregnancy to encourage attitudes and service models that are inclusive of fathers, regardless of whether they are partnered with the mother.
• Maternity services should work with the voluntary sector in order to enable young fathers-to-be to be better prepared for becoming a father, for example by introducing Expectant Fathers Programmes and peer-led ante-natal classes, such as the Fatherhood Institute's Hit the Ground Crawling programme.^6^
• Maternity services should work with their local children's centres to offer a paediatric first aid course to all young dads.
• Health visiting services should investigate creating a memorandum of understanding with their local children's centres to share their knowledge of local need and ensure new families with support needs are always registered by health professionals with a local children's centre.^7^

## Young dads and children's centres

Sure Start children's centres are crucial early years’ settings that operate at the heart of local communities. Many have targets for engaging with dads. However, there are no data on how many young dads they come into contact with. Some children's centres offer specific activities for fathers, such as paediatric first aid, parenting courses, football tournaments and dad and baby days out. However, in many cases, children's centres will only ever come into contact with the mother and child. Children's centres also refer fathers to parenting programmes, such as the Barnardo's BabyFather Initiative's Fatherhood Programme.^8^

## Good practice example

Barnardo's BabyFather initiative works with children's centres in London to provide training and consultancy services to professionals supporting children, families, men, fathers and male carers. The Fatherhood Training Programme is designed to increase professionals’ understanding and confidence, providing practical skills while looking at social policy, legislation and the theory of fatherhood in the community. Children's centres in London refer fathers to the BabyFather Initiative's Fatherhood Parenting Programme. This ten week accredited course enables dads to reflect on and evaluate their role and importance as fathers, to demonstrate increased confidence to parent and to understand the various stages of child development. By working in partnership with the voluntary sector, children's centres have become more aware of young fathers’ needs in their local areas, and have been able to offer them more targeted support in the children's centre setting.

## Case study 2: Luke's journey

Luke met Amy at school and they started dating. He sat his GCSE (General Certificate of Secondary Education) exams and did well. When Luke left school to go to university, he and Amy broke up. In his first term at university, Luke found out he was going to be a father. He was shocked, and initially did not want Amy to have the child, but then became supportive of her decision. He attended all of the hospital appointments during the pregnancy, and decided to leave university to get a job as a Sales Advisor.

Luke and Amy's relationship broke down just before the birth. The baby was born but Amy did not inform Luke. This caused animosity between them and he did not see his baby until he was four weeks’ old, which he found very difficult. Luke and Amy started talking again and she let him see the baby. Soon he started caring for him several days a week and at one point looked after his son full-time for two months while Amy had a break.

Luke was studying for an NVQ (National Vocational Qualification) in child care. At this point, he heard about Barnardo's BabyFather initiative through his local children's centre. He was supported to maintain a positive relationship with Amy and learnt parenting strategies that helped him support his son. Luke's son is now five years old. Luke has a different life now and different friends, but ambition for the future. He is volunteering for the BabyFather initiative to share his experience with other dads.

## Recommendations for children's centres

• Children's centres should adopt a culture of asking about the father whenever they have contact with a mother, and keep a record of the young dads that do attend.
• Children's centres should refer on to voluntary sector services in the local area that are able to offer specialist support to young dads.
• Children's centres should not assume that young dads will want to attend the same groups as young mums, but instead introduce targeted activities, such as dad and baby days out and sports events.
• Children's centres should introduce a weekly drop-in clinic for young dads to address parenting concerns. Staff at the clinic could also signpost the dads to additional support, for example with housing or employment.

## Young dads in school

The total number of under-eighteen conceptions in England and Wales was 27,834 in 2012 (ONS, 2012). However there are no statistics to show the number of teenage fathers, and local authorities do not collect data on the number of young fathers in school. Boys who become fathers as teenagers have been found to be three times more likely than non-fathers to fail to complete secondary education, and also tend to be far less satisfied with their educational experience (Fatherhood Institute, 2010). Yet research has also shown that school attendance can act as a protective factor, and the school years may be the optimal time to address a range of risk behaviours in young dads (Weinman *et al.*, 2007). Teenage fathers are more likely to be not in education, employment or training (NEET) than their peers. Therefore school and local authority NEET prevention strategies must consider the specific needs of teenage fathers.

## Good practice example

Leeds City Council Children's Services Department has a learning mentor^9^ who supports teenage fathers in school. Typically, a young dad will have an initial meeting with the learning mentor to look at his timetable to see when he might need to be away from class to attend antenatal appointments. Father support includes liaising with the father's family and a range of professionals, and a weekly after school group for young dads to offer peer support and address any education issues. The learning mentor discusses what each young father needs in terms of continuing his education, planning a career and his role as a parent. Often the learning mentor will work to get young dads back on track where they have not been engaged with education for some time. For these young men, becoming a father is a motivating factor for re-engaging, and there is evidence that young fathers benefit greatly from one-to-one emotional support provided by services such as this (Neale and Lau Clayton, 2011).

## Case study 3: Dominic's journey

Dominic became a father when he was sixteen. He split up with his partner shortly after the birth. Dominic was doing well at school but felt that he could not go to university because he needed to provide financially for his child. He was living at home with his mum and dad.

Dominic was introduced to a learning mentor at school, who helped him talk through his concerns about becoming a father. The learning mentor supported Dominic to adjust to being defined as a father as well as a young man, and having less freedom than his peers, which had led to him feeling low. Dominic needed guidance in the early stages to stick with the baby and get on with the mother in order to develop a relationship with his child.

Dominic started studying part-time for a degree whilst working in an office job. He struggled with feeling trapped in a job he did not want just to pay the bills, but supporting his child financially was his main priority. He still had ambitions for his life but did not know if he would ever be able to fulfil them. Dominic had always wanted to go to university but had no knowledge of how to do so while also looking after his son. His learning mentor helped him and as a result he enrolled on a part-time degree programme and is doing very well.

## Recommendations for schools

• All schools should authorise absence for young dads to attend health appointments and allocate a member of staff to support each young father.
• Schools need to intervene early, as soon as a young man finds out he is going to be a dad. The transition to fatherhood is a time when young men experience an increased sense of responsibility, greater ambition to achieve, as well as the need to provide financially. As such, the point of entry into fatherhood can be when young men are most likely to engage in education, training or employment (Ross *et al.*, 2010).
• The government should introduce parenting education for all secondary school pupils. This would support the government's aims of normalising support for parents, as well as supporting young parents who are still in school to gain parenting skills.

## Young dads and housing support

Many young fathers present to their local authority as homeless when they are no longer able to stay with their parents, or no longer able to afford to live independently. Homelessness legislation in England states that councils should consider anyone to be in priority need if they are responsible for dependent children who normally live with them (or would do were accommodation available).^10^

When young fathers have their children living with them on a part-time basis, decisions can become complicated, and the child's best interests are not necessarily considered in the housing allocation process. Furthermore, under Local Housing Allowance rules, single people aged under thirty-five who either do not have children or are not primary carers are normally assumed to be living in shared accommodation.^11^ Children visiting or staying with young fathers in this situation could be put at risk, and many young fathers will be forced to find an alternative location at which to see their children.

The young fathers’ journeys reported here show that housing is central to their ability to look after and build a relationship with their children. Taking a whole family approach to enable young parents to establish their own households would make a significant difference to parent and child wellbeing. Young parents in couple relationships are often forced to live separately and decisions about where the baby stays are made by the maternal household in particular (Neale and Lau Clayton, 2014).

## Good practice example

Leeds Housing Concern^12^ is a charity which responds to the needs of vulnerable homeless and disadvantaged young people and seeks to promote their greater social inclusion. Its specialist Young Person's Project (YPP) provides fully furnished single bed accommodation and shared housing in one and two-bedroomed houses for young people aged between sixteen and twenty-five. All young people are allocated a key worker who will agree and coordinate a tailored support package. The key worker meets regularly with them on an individual basis and makes referrals as required to other agencies, such as health, counselling, psychiatric, detox and advocacy services. The YPP also offers young people advice on benefit entitlement, job seeking, pregnancy and parenting. Referrals to the YPP are made by a wide range of voluntary and statutory organisations, and young people may use the service for up to six months.

## Case study 4: Darren's journey

Darren met Emily at school. He was fifteen when he found out he was going to be a dad. After the birth, Darren, Emily and the baby were living with Darren's parents. Darren and his mum argued about how to look after the baby, and Darren's and Emily's relationship suffered due to the lack of space.

A friend told Darren about the Young Person's Housing Project run by a local charity. Darren moved into the project's accommodation with his friend and redecorated to prepare for Emily and the baby. Darren had to learn how to live independently for the first time and how to manage his rent and bills. The charity also helped Darren to access other services like the Job Centre.

After eighteen months sharing with a friend, Darren, Emily and the baby moved into a two bedroomed council house of their own. The house needed redecorating and refurnishing, so Darren used his benefit payments and a loan from his dad to do this. Refurnishing was required by social services and it left them in financial difficulty as they were already in debt. Having to use their benefits on furnishings meant drastically reducing their food and electricity bills for a short period.

Darren's son is now six years old and doing well at school. Darren is unemployed due to ill health but feels his role as provider is highly important and a part of responsible fatherhood duties. Darren views fatherhood as a wholly rewarding experience and identifies many positive aspects to being a young parent, having learnt important life skills through the Young Person's Housing Project. Darren has high aspirations for his son's future. Despite not going to university himself, he hopes that his son will enter higher education one day.

## Recommendations for local authority housing services

• Housing should not be a barrier to a young father's ability to take care of his child. Local authorities should ensure that young fathers as well as mothers are classified as ‘priority need’ and allocated appropriate housing accordingly. In particular, they should consider the best interests of the child when allocating housing to young fathers, and recognise the safeguarding implications of placing a young father in shared accommodation.
• Housing officers should record and identify young fathers, referring them on to voluntary sector support where available.

## Young dads in custody

There are no data to show the number of young fathers in custody in England. However, the Ministry of Justice estimates that 53 per cent of men in prison have a child, and that there are approximately 200,000 children of male prisoners in England and Wales (Prison Reform Trust, 2014). In 2011, there were 8,089 young men in custody aged eighteen to twenty, and a further 1,523 young men under the age of eighteen (Ministry of Justice, 2012). The Prison Reform Trust (2014: 32) notes that:

No-one routinely monitors the parental status of prisoners in the UK or systematically identifies children of prisoners, where they live, or which services they are accessing; where this information is collected, it is patchy and not always shared. Prison governors receive no specific funding to meet the costs of family support work, parenting courses, family visitor centres or supervised play areas. This means any family provision must come from a governor's already stretched and shrinking general prison budget.

Many of the services available to young fathers in the community are not accessible for fathers in prison, including online information. Instead, practitioners must visit them where they are. Cuts to family liaison and social work provision in young offenders’ institutions have made this increasingly challenging. Research has shown that young offenders who are fathers are more likely to engage in parenting programmes while in prison than following release (Meek, 2007). In addition, interventions that link young fathers to employment and community services as part of resettlement plans have been shown to reduce the risk of reoffending (Social Exclusion Unit, 2002).

## Good practice example

Safe Ground^13^ works to reduce the risk of offending and reoffending based on a continually developing understanding of the origins and impact of crime and a commitment to empowering people to change, whether in prison or the community. Their prison programmes, Family Man and Fathers Inside, use drama, group work and communication skills to strengthen family ties, develop critical thinking and engage reluctant learners. Since 2003, Fathers Inside has been delivered in thirty-seven prisons with over 2,000 men graduating from the programme and almost 5,000 qualifications awarded. The fundamental aim of Fathers Inside is to help prisoners contribute to society by teaching them how to support their children's education and upbringing, while they are in custody and after release.

## Case study 5: Jon's journey

When Jon found out he was going to be a dad, he was no longer in a relationship with the mother of the baby. Although he wanted to support the mother during her pregnancy, he became de-motivated by being unsure what support he could provide and by his poor relationship with her family. He had left school, but, like most of his peer group, was unable to find employment. He ended up in a young offender's institution, serving a community order for actual bodily harm and criminal damage. The mother of his baby was angry with him for being ‘irresponsible’ and having ‘anger management’ issues.

Jon was unable to contact his child's mother after her initial refusal to visit him, and was struggling with conflicted feelings about wanting to maintain contact with his child, and feeling unable to adjust to being a father.

At age nineteen, Jon was referred to Safe Ground's Fathers Inside programme by his offender supervisor, who thought it would help to meet Jon's needs and enable him to progress through his sentence plan.

Jon took part in Fathers Inside reluctantly. He was not convinced it would be of any use at all. Despite his initial resistance, Jon met other men with whom he related well, and he started to develop new skills through participating in the course's drama-based activities. Jon attended the ‘What Next’ session as part of the Fathers Inside course. ‘What Next’ gives Jon an opportunity to access services that can provide support to him and his family.

Jon also identified organisations that could help him to become a more engaged parent. A structured action plan encouraged Jon to plan for his future both while he was in prison and upon release. Through the letter-writing exercises, Jon re-established contact with his child's mother. At the end of the Fathers Inside course, Jon performed in a presentation to family members and prison staff. Although the mother of his baby did not come to the presentation, she did send him a letter of encouragement and a photo of the baby for the first time.

Jon is approaching the end of his time in prison. The Fathers Inside family support worker was able to follow up referrals made for Jon at the ‘What Next’ session, and as a result he has made contact with a parenting advisory agency in his local area. Jon's prison officer and offender supervisor are really keen to support him as he has made so much progress. He has passed five GCSEs in addition to making plans to attend college to do a catering course on release. He has begun to build a friendly relationship with the mother of his child, who now says she is willing to bring the child along to the prison's next family visit with a view to Jon keeping in contact upon release.

## Policy implications

• Systematic collection of data on the number of fathers in custody should be carried out routinely and the data made available to services seeking to support fathers in custody.
• Tailored parenting programmes should be available to all fathers in the secure estate, taking a holistic family support approach.
• Resettlement plans should include signposting to relevant support services for fathers, including at children's centres.
• Currently mothers under the age of eighteen are not allowed to visit a partner in prison without an adult present. This restricts the amount of contact a young father has with both his partner and his child and should be reviewed.
• Information for prisoners about how to make contact and arrange visits with the mother and their child should include advice on the impact on future relationships and their children's outcomes of not maintaining contact.

## Conclusion

These young fathers’ journeys show young men adjusting to the reality of fatherhood at an age when they would otherwise be making critical choices about what to do with their lives as individuals. The journeys demonstrate the role of the family voluntary sector in supporting young fathers at key points to help them make a successful transition to fatherhood. They also reveal the value of co-ordinated support across voluntary and statutory provision. The best practice examples show how services can tailor the support they offer, and partner with each other, to meet young fathers’ support needs. A lead professional in each local authority with an overview of the young fathers in the area, and the support available to them, would enable a targeted approach. Commissioners and service providers alike must recognise that supporting positive child outcomes must include supporting young fathers to take an active role in their children's lives. The most effective time to engage a young father is during the pregnancy, therefore early intervention strategies to engage young mothers must also engage young fathers. This encourages a lifelong commitment to fatherhood and better outcomes for both young fathers and their children.

Statutory and voluntary services do not work with families in isolation. Schools, health professionals, children's centres, housing services, prisons and the voluntary sector must share information and refer on to each other to ensure that all young fathers access the support they need. However, in order for this to be possible, those services must challenge negative assumptions about the role of young fathers consistently and emphatically.

## Notes

1 For more information, see www.fatherhoodinstitute.org/2009/about-the-think-fathers-campaign/ [accessed 29.12.2014].

2 For more information, see www.gov.uk/help-with-childcare-costs/free-childcare-and-education-for-2-to-4-year-olds [accessed 31.03.15].

3 For more information, see www.canparent.org.uk/ [accessed 31.03.15].

4 An evaluation report of the Department for Education's relationship support interventions is available at www.gov.uk/government/publications/relationship-support-interventions-evaluation [accessed 31.03.15].

5 For more information, see www.workingwithmen.org/index.php?option = com_content&view = article&id = 72&Itemid = 68 [accessed 29.12.2014].

6 For more information, see www.fatherhoodinstitute.org/hit-the-ground-crawling [accessed 29.12.2014].

7 For example, Northumberland Sure Start Children's Centres and Northumbria Healthcare NHS foundation Trust Health Visiting Service launched a Memorandum of Understanding (MOU) in July 2012 to ensure a shared understanding of local community need, enabling them to target their resources effectively to meet these.

8 For more information, see www.barnardos.org.uk/babyfather.htm [accessed 29.12.2014].

9 Leeds Learning mentors were partners in an ESRC funded tracking study of young fatherhood conducted under the Timescapes research programme led by the University of Leeds, 2010–12.

10 For more information, see http://england.shelter.org.uk/get_advice/homelessness/homelessness_-_an_introduction/legal_definition_of_homelessness [accessed 29.12.2014].

11 For more information, see DWP Local Housing Allowance Guidance www.dwp.gov.uk/local-authority-staff/housing-benefit/claims-processing/local-housing-allowance/ [accessed 29.12.2014].

12 For more information, see Leeds Housing Concern www.leedshc.org.uk/en/scheme/lhc-ypp.aspx [accessed 29.12.14].

13 For more information, see www.safeground.org.uk/gallery/family-man-and-fathers-inside/ [accessed 29.12.2014].
